# Roles of *NR1I3* and *NR1H4* polymorphisms in the susceptibility to antituberculosis drug-induced liver injury in China: a case‒control study

**DOI:** 10.3389/fgene.2024.1428319

**Published:** 2024-10-23

**Authors:** Xiaoyan Xu, Ruina Chen, Lihuan Lu, Jingru Cheng, Xiaomin He, Hongqiu Pan, Meiling Zhang, Honggang Yi, Shaowen Tang

**Affiliations:** ^1^ Department of Infectious Disease Control and Prevention, Changshu Center for Disease Control and Prevention, Suzhou, China; ^2^ Department of Epidemiology and Biostatistics, School of Public Health, Nanjing Medical University, Nanjing, China; ^3^ Department of Tuberculosis, The Second People’s Hospital of Changshu, Changshu, China; ^4^ Department of Infectious Disease, The People’s Hospital of Taixing, Taixing, China; ^5^ Department of Tuberculosis, The Third People’s Hospital of Zhenjiang Affiliated to Jiangsu University, Zhenjiang, China; ^6^ Department of Infectious Disease, The Jurong Hospital Affiliated to Jiangsu University, Jurong, China

**Keywords:** antituberculosis drug-induced liver injury, nuclear receptor subfamily 1 group I member 3, nuclear receptor subfamily 1 group H member 4, genetic polymorphisms, case-control study

## Abstract

**Objective:**

The pathogenesis of antituberculosis drug-induced liver injury (AT-DILI) remains largely unknown. The current investigation aimed to determine the genetic contribution of the nuclear receptor subfamily 1 Group I member 3 (*NR1I3*) and nuclear receptor subfamily 1 Group H member 4 (*NR1H4*) genes to the risk of AT-DILI in the Chinese population.

**Methods:**

A 1:4 matched case‒control study was conducted, and five single nucleotide polymorphisms (SNPs) in the *NR1I3* and *NR1H4* genes were detected and assessed. Utilizing a multivariate conditional logistic regression model, the effects of haplotype and genotype on the risk of AT-DILI were examined. Extended subgroup analysis was carried out based on sex. The distribution of the peak value of serum liver enzymes also compared among different genotypes.

**Results:**

224 AT-DILI cases and 896 controls were included in this study. No significant difference was observed in genotypes or haplotypes frequencies between AT-DILI cases and controls. However, comparisons of liver function indicators revealed significant differences in the peak values of alanine aminotransferase (ALT), aspartate aminotransferase (AST) and total bilirubin (TBil) among patients with different genotypes of *NR1H4* rs56163822 (GG vs. GT vs. TT, 27.1 U/L vs. 26.0 U/L vs. 23.0 U/L, *p* = 0.020; 34.0 U/L vs. 31.0 U/L vs. 30.6 U/L, *p* = 0.008; 15.5 μmol/L vs. 15.0 μmol/L vs. 13.7 μmol/L, *p* = 0.029, respectively), as well as in the peak values of ALT and AST among male patients with different genotypes of *NR1H4* rs56163822 (29.0 U/L vs. 26.9 U/L vs. 22.6 U/L, *p* = 0.002; 34.0 U/L vs. 32.0 U/L vs. 30.5 U/L, *p* = 0.019, respectively).

**Conclusion:**

Based on this 1:4 individual-matched case‒control study, the SNP rs56163822 in the *NR1H4* gene may be linked to the susceptibility to AT-DILI in Chinese patients receiving anti-TB treatment. Further studies in larger varied populations are needed to validate our findings.

## Introduction

Tuberculosis (TB) is treated in China with the regimen recommended by the World Health Organization (WHO), which includes a combined therapy of isoniazid (INH), rifampicin (RIF), pyrazinamide, ethambutol and/or streptomycin for 6 months (WHO, 2017). This is the most commonly used standard anti-TB treatment; however, long-term use of multidrug combination therapy may lead to a variety of adverse drug reactions (ADRs) in patients (Arbex et al., 2010). Among all kinds of ADRs, anti-TB drug-induced liver injury (AT-DILI) exhibits a comparatively higher occurrence rate (Wang et al., 2022). At present, the exact mechanism of AT-DILI is not fully understood, and there are many factors affecting the occurrence of AT-DILI, including social, environmental, and genetic factors, such as age, female sex, concomitant hepatitis C, and gene polymorphisms (Gaude et al., 2015; Ding et al., 2017; Zheng et al., 2020; Yang et al., 2019a).

In recent years, animal experiments have shown that the accumulation of protoporphyrin Ⅸ (PPIX) in hepatocytes may be a potential new mechanism of AT-DILI (Sachar et al., 2016a). Pregnane X receptor (PXR), a significant member of the nuclear receptor family, functions as a transcriptional regulator of cytochrome P450 (CYP450) 3A4 (CYP3A4). It binds to the response element of the CYP3A4 gene’s promoter and operates in a heterodimeric form with the retinoid X receptor (RXR). RIF-mediated activation of PXR induces aminolevulinic synthase-1 (ALAS1) expression by upregulating CYP450 through the PXR pathway (Sachar et al., 2016b; Lyoumi et al., 2013; He et al., 2017). Animal experimental studies have shown that in mice, human-derived PXR can upregulate ALAS1 expression by binding to the drug response enhancer sequence in the flanking region of ALAS1 (Podvinec et al., 2004; Fraser et al., 2003). When exposed to agents that stimulate CYP450 and other drug-metabolizing enzymes, ALAS1 transcription is upregulated, the rate of haem biosynthesis is limited, and PPIX synthesis is increased, leading to hepatic accumulation and liver injury. In addition, studies have suggested that the metabolites of INH, hydrazine and acetyl hydrazine, are related to drug-induced liver injury (DILI). Recent animal experiments have suggested that INH can induce the expression of ALAS1 and reduce that of ferrochelatase (FECH) in mice, resulting in a decrease in the synthesis of haem from PPIX and Fe^2+^ and an increase in PPIX levels *in vivo* (Sachar et al., 2016b).

In the PXR pathway, heat shock protein 90 (HSP90) serves as a molecular chaperone. The cytosolic constitutive androstane receptor (CAR, encoded by the *NR1I3* gene) interacts with PXR (Squires et al., 2004; Moffatt et al., 2008) and is also involved in regulating HSP90 activity (Brychzy et al., 2003). The CAR is highly expressed in the liver and shows an overlapping set of target genes with PXR in response to potentially harmful chemicals (Swales and Negishi, 2004; Timsit and Negishi, 2007). Overexpression of CAR in the presence of PXR leads to an elevation in the cytoplasmic concentrations of the PXR-CAR-HSP90 complex, which is retained in the cytoplasm of HepG2 cells (Squires et al., 2004). Upon binding to its ligand, PXR undergoes a conformational alteration, prompting its association with RXR to generate PXR/RXR heterodimers. These heterodimers then transfer to the nucleus and recruit transcriptional coactivators or corepressors to form complexes. These complexes specifically recognize and bind to response elements situated within the proximal promoter regions of target genes, thereby modulating their transcriptional activity and expression patterns (Tian, 2013). However, PXR/RXR heterodimers activate the xenobiotic-responsive enhancer module in response to RIF (Goodwin et al., 1999), thereby affecting the body function. Normally, the PXR-HSP90-CAR complex is located in the cytoplasm, however, the RIF-INH combination triggers the dissociation of this complex, the translocation of PXR to mitochondria via interaction with RXR. Subsequently, the ligand-bound PXR-RXR complex binds to specific DNA response elements, leading to the transcription of ALAS1 and CYP450. Direct transcription of ALAS1 can be activated by RIF or INH, or it can be downregulated by negative feedback disinhibition triggered by the incorporation of haem into CYP450 apolipoproteins (Lyoumi et al., 2013). This dual mechanism may be the cause for the accumulation of the endogenous hepatotoxicant PPIX, which leads to AT-DILI. However, few studies have investigated the association between NR1I3 gene polymorphisms and AT-DILI, and the results are negative (Wang et al., 2019). Therefore, we hoped to verify this result by expanding the sample size.

Research suggest that RIF-induced liver injury, steatosis, and cholestasis are associated with farnesoid X receptor (FXR, encoded by the NR1H4 gene) dysfunction and altered bile acid (BA) metabolism, and that the JNK signaling pathway is partially implicated in this injury. Therefore, FXR may represent a novel therapeutic target for addressing DILI (Zhou et al., 2024; Ezhilarasan, 2023). Wen et al. found that HRZE may upregulate FXR with short-term administration, but more prolonged treatment appears to suppress FXR function, resulting in hepatic BA accumulation, suggestive of hepatotoxicity (Wen et al., 2022). INH + RIF administration decreased the expression of BA transporters such as the bile salt export pump and multidrug resistance-associated protein 2, and induced liver injury through sirtuin 1 and FXR pathway (Ezhilarasan, 2023). PZA induces cholestatic liver injury by FXR inhibition, leading to dysfunction in BA synthesis and transport (Guo et al., 2016). Pharmacological studies have provided compelling evidence that FXR exerts protective effects against diverse etiologies of DILI through its anticholestatic, anti-adipogenic, antioxidant, anti-inflammatory, and antifibrotic effects. Further research has demonstrated that FXR agonists potently mitigate hepatotoxicity and foster liver regeneration in mice models with liver injury (Carino et al., 2018). However, the association between FXR gene polymorphism and AT-DILI has not been studied.

Therefore, the current study endeavors to elucidate the genetic predisposition of *NR1I3* and *NR1H4* genes in the risk of AT-DILI among the Chinese population, employing single-nucleotide polymorphisms (SNPs) as markers and adopting an individual-based matched case-control study design.

## Methods

### Study design and setting

The Ethics Committee of Nanjing Medical University granted approval for this research. Between May 2016 and December 2021, patients receiving anti-TB treatment were recruited from the outpatient departments of four designated infectious disease hospitals (Jurong People’s Hospital, Taixing People’s Hospital, The Second People’s Hospital of Changshu and The Third People’s Hospital of Zhenjiang) in Jiangsu Province, covering both urban and rural areas. This study was conducted according to STROBE checklist for case-control studies (Supplementary Table S1).

### Patients and clinical criteria

The inclusion criteria for this study encompassed patients who: (1) had a newly diagnosed TB; (2) were poised to undergo anti-TB therapy; (3) could actively undergo regular liver function tests during the first 2 months of treatment and had at least 3 cycles of liver function test information available; and (4) provided voluntary informed consent. The exclusion criteria for this study: (1) TB patients exhibiting abnormal baseline liver function indicators, such as alanine aminotransferase (ALT), aspartate aminotransferase (AST), and total bilirubin (TBil) levels; (2) with mental illness; (3) with severe comorbidities.

### AT-DILI case and control definition and selection

The diagnosis of AT-DILI was confirmed in accordance with the Chinese guidelines (Yu et al., 2017), ALT is greater than or equal to 3 upper limit of normal (ULN) and/or TBil is greater than or equal to 2 ULN; or AST, ALP and TBil increased at the same time, and at least one item is greater than or equal to 2 ULN. Three distinct patterns of injury were delineated, namely, ‘hepatocellular’, ‘mixed’, or ‘cholestatic’, based on the R ratio. The R ratio was determined by employing the ratio of the ALT level to the alkaline phosphatase (ALP) level, both values being normalized by their respective local upper limits of normal (ULN). Specifically, the calculation was conducted as follows: R = (ALT value/ALT ULN)/(ALP value/ALP ULN). An R ratio > 5 indicated hepatocellular injury; that of < 2 indicated cholestatic injury; and an R ratio between 2 and 5 indicated mixed injury. Additionally, causality assessment was conducted in accordance with the International Consensus Criteria (Roussel Uclaf Causality Assessment Method, RUCAM) (Danan and Teschke, 2015), and all patients with possible (score 3–5), probable (score 6–8), or highly probable (score > 8) were included in the study. Furthermore, the severity of liver injury was categorized as mild (ALT < 5 ULN), moderate (ALT 5–10 ULN), or severe (ALT > 10 ULN), adhering to the WHO toxicity classification standards (Tostmann et al., 2008).

Patients who met the diagnostic criteria for AT-DILI were categorized into the case group, while those whose serum liver function tests remained consistently normal throughout anti-TB treatment were deemed candidate controls. For each AT-DILI patient, four control individuals of the same sex and within a 5-year age range were randomly selected.

### Variables

In this study, the prevalence of AT-DILI was selected as the outcome variable. Liver disease history is defined as having had a liver condition in the past. Similarly, smoking history is defined as having smoked at least two cigarettes per day for the past 2 weeks. In relation to alcohol intake, drink history is defined as having drunk at least 50 mL of alcohol per day for the past 2 weeks. Furthermore, hepatoprotectant use refers to the use of hepatoprotectant during the course of treatment.

### Data sources/measurement

Prior to the initiation of anti-TB treatment, liver function tests (LFTs) were administered. During the initial 2 months of anti-TB therapy, patients underwent scheduled outpatient follow-ups and underwent LFTs every 2 weeks at the designated hospitals, adhering to the local free-TB service policy. In the event that patients exhibited symptoms indicative of potential hepatitis (such as anorexia, nausea, vomiting, malaise, or tea-coloured urine), they were instructed to promptly visit the designated hospital for immediate LFTs (Chen et al., 2019; Yang et al., 2019b). Despite the absence of any manifestations suggesting drug-induced hepatitis, patients were counseled to undergo regular LFTs at a biweekly interval. Residual blood samples from the LFTs of each patient were retained for further analysis. All recruited TB patients were closely monitored throughout their treatment regimen until completion.

### SNP selection and genotyping

The leftover blood sample from an LFT was utilized for the extraction of genomic DNA. The Han Chinese individuals in Beijing (CHB) dataset of the 1000 Genomes Project database served as the source for downloading all eligible SNPs in the *NR1I3* and *NR1H4* gene regions, along with regions 2 kb upstream and downstream of the genes. Utilizing the Haploview software version 4.2 from Broad Institute in Cambridge, MA, SNPs were chosen based on specific criteria: (1) a minor allele frequency (MAF) ≥ 10% and (2) an r^2^ of pairwise linkage disequilibrium ≥ 0.8 (Eberle et al., 2006). Furthermore, HaploReg version 4.1 was used to maximally select potentially functional SNPs. Finally, a total of five SNPs, rs2307424, rs2502805, rs10157822, and rs11584174 in the *NR1I3* gene and rs56163822 in the *NR1H4* gene, were identified for the genotyping using TaqMan allelic discrimination technology (Supplementary Table S2). To ensure unbiased genotyping, the case or control status was blinded during the process. Additionally, over 10% of the samples were randomly selected for duplicate genotyping using the same assay, resulting in a 100% accuracy rate.

### Statistical analysis

Continuous variables are described as the means ± standard deviations or as medians with interquartile ranges (IQRs), and the independent-samples t-test or the Wilcoxon rank-sum test were used to analyse differences between groups. Categorized variables are represented as the count of patients and a percentage and were analysed using a conditional logistic regression model. The chi-square goodness-of-fit test was conducted to assess Hardy-Weinberg Equilibrium (HWE) in the control group. Utilizing the multivariate conditional logistic regression model, odds ratios (ORs) were computed, along with their respective 95% confidence intervals (CIs). The liver disease history and smoking history were adjusted. Three genetic models (dominant, recessive, and additive) were constructed to comprehensively analyse the effects of the SNPs on the risk of AT-DILI. Haplotype blocks were constructed using the HaploView 4.2 software, considering the linkage disequilibrium (LD) among SNPs. The haplo.stats package in the R software was employed to estimate the haplotype of each individual based on the observations. Subgroup analyses were performed, each according to sex. For the association analysis of gene polymorphisms, false discovery rate (FDR) correction was implemented to correct for multiple comparisons. The genetic variants that could affect different gene expression were identified via online expression quantitative trait loci (eQTL) analysis from GETx-Portal (https://www.gtexportal.org/home/). All the analyses were conducted using the R software for Windows version 4.2.2. Statistical significance was inferred when the two-tailed *p*-value < 0.05.

## Results

### Baseline and clinical characteristics

A total of 1120 TB patients (224 AT-DILI cases and 896 controls) were included in the study. There were 749 patients treated with the 2HREZ/4HR regimen, 149 with the 3HREZ/9HR regimen, and 222 with other treatment regimens. The chi-square test results show that there was no difference in treatment regimens between the two groups (*p* > 0.05). Among the 224 AT-DILI cases, 124 (55.4%) were judged as probable, 131 (58.5%) had mild liver injury, and 147 (65.6%) were classified as the hepatocellular type of liver injury. Table 1 summarized the baseline demographic and clinical characteristics of the patients and matched controls. No significant differences were observed in the distributions of age, sex, drinking history, hepatoprotectant use or baseline hepatic biochemical parameters between the case groups and control groups (*p* > 0.05). The proportions of AT-DILI cases with liver disease or smoking history exceeded those of controls. During the course of anti-TB therapy, the peak levels of AST, ALT, TBil, direct bilirubin (DBil) and ALP in the AT-DILI cases were significantly elevated compared to those in the controls (*p* < 0.0001).

**TABLE 1:** Characteristics of patients in AT-DILI cases and controls.

Characteristics	AT-DILI cases (N = 224)	Controls (N = 896)	*p*-value
Sex (male/female)	164/60	656/240	—
Age (years)^a^	50.1 ± 17.7	50.7 ± 18.2	0.651^*^
Liver disease history (with/without)	49/175	139/757	0.019^†^
Smoke history (yes/not)	29/195	78/818	0.041^†^
Drink history (yes/not)	10/214	39/857	0.941^†^
Hepatoprotectant (use/not use)	115/109	469/427	0.756^†^
Baseline value			
ALT(U/L)^b^	15.0(9.0–21.7)	13.7(9.0–20.0)	0.409^‡^
AST(U/L)^b^	21.0(15.0–25.0)	20.0(16.0–24.0)	0.391^‡^
TBIL(μmol/L)^b^	10.1(7.9–12.9)	10.0(7.5–13.1)	0.815^‡^
DBIL(μmol/L)^b^	3.4(2.3–4.0)	3.3(2.3–4.4)	0.447^‡^
ALP(U/L)^b^	83.0(67.4–90.0)	81.1(63.7–93.0)	0.218^‡^
During treatment (peak value)			
ALT(U/L)^b^	173.0(132.5–278.8)	23.0(15.0–32.0)	<0.0001^‡^
AST(U/L)^b^	119.0(70.5–200.0)	29.0(23.0–39.0)	<0.0001^‡^
TBIL(μmol/L)^b^	19.4(14.1–25.1)	14.0(10.5–19.0)	<0.0001^‡^
DBIL(μmol/L)^b^	7.3(5.1–10.9)	4.7(3.4–6.6)	<0.0001^‡^
ALP(U/L)^b^	121.0(87.0–148.0)	91.1(76.0–114.8)	<0.0001^‡^

AT-DILI, antituberculosis drug-induced liver injury; ALT, alanine transaminase; AST, aspartate transaminase; TBil, total bilirubin; DBil, direct bilirubin; ALP, alkaline phosphatase. Normal range: ALT<40U/L; AST<40U/L; TBil<21 μmol/L; DBil<6.9 μmol/L; ALP<130U/L.

^a^ Values are presented the mean ± standard deviations.

^b^ Values are presented as median (interquartile range).

*Independent-samples t-test.

^†^Conditional logistic regression model analysis.

^‡^Wilcoxon rank sum test.

### Genotype analysis

Multivariate conditional logistic regression analysis exhibited that patients carrying the CT genotype of rs2502805 were at a lower risk of AT-DILI than those with the CC genotype (adjusted OR = 0.675, 95% CI: 0.488–0.934, *p* = 0.018), and the risk of AT-DILI in patients with CT + TT genotypes was lower than that in patients with the CC genotype (adjusted OR = 0.705, 95% CI: 0.521–0.952, *p* = 0.023) according to the dominant model. However, after FDR correction, the difference between the groups was no longer statistically significant (*p* > 0.05) (Table 2).

**TABLE 2:** Genotypes distribution of *NR1I3* and *NR1H4* genes in two groups and the risk of AT-DILI.

Gene	SNPs	AT-DILI cases (N = 224)		Controls (N = 896)		OR (95%CI)^a^	*p*-value	*p*-value^*§*^	Model	OR (95%CI)^a^	*p*-value	*p*-value^*§*^
		n	%	n	%							
*NR1I3*	rs10157822(T>C)											
	TT	32	14.3	155	17.3	1.000			Dom	1.287(0.851–1.948)	0.232	
	TC	107	47.8	436	48.7	1.220(0.787–1.892)	0.374		Rec	1.193(0.874–1.628)	0.266	
	CC	85	37.9	305	34.0	1.383(0.878–2.179)	0.162		Add	1.167(0.940–1.448)	0.161	
	rs11584174(C>T)											
	CC	121	54.0	517	57.7	1.000			Dom	1.170(0.866–1.581)	0.307	
	CT	83	37.1	301	33.6	1.181(0.859–1.623)	0.305		Rec	1.047(0.616–1.782)	0.865	
	TT	20	8.9	78	8.7	1.122(0.648–1.943)	0.681		Add	1.106(0.879–1.390)	0.390	
	rs2307424(G>A)											
	GG	63	28.1	261	29.1	1.000			Dom	1.078(0.778–1.493)	0.653	
	GA	104	46.4	411	45.9	1.079(0.760–1.533)	0.671		Rec	1.026(0.733–1.437)	0.880	
	AA	57	25.5	224	25.0	1.075(0.721–1.604)	0.722		Add	1.038(0.851–1.266)	0.714	
	rs2502805(C>T)											
	CC	106	47.3	348	38.8	1.000			Dom	0.705(0.521–0.952)	0.023	0.092
	CT	88	39.3	429	47.9	0.675(0.488–0.934)	0.018	0.072	Rec	0.968(0.627–1.494)	0.883	
	TT	30	13.4	119	13.3	0.801(0.506–1.268)	0.344		Add	0.826(0.663–1.030)	0.090	
*NR1H4*	rs56163822(G>T)											
	GG	125	55.8	449	50.1	1.000			Dom	0.769(0.568–1.042)	0.090	
	GT	57	25.4	266	29.7	0.737(0.516–1.052)	0.092		Rec	0.912(0.615–1.353)	0.648	
	TT	42	18.8	181	20.2	0.823(0.545–1.241)	0.352		Add	0.873(0.716–1.066)	0.183	

*NR1I3*, nuclear receptor subfamily 1 group I member 3; *NR1H4*, nuclear receptor subfamily 1 group H member 4; AT-DILI, antituberculosis drug-induced liver Injury; SNPs, single nucleotide polymorphisms; OR, odds ratio; 95% CI, 95% confidence interval; Dom, dominant model; Rec, recessive model; Add, additive model.

^a^ Conditional logistic regression analysis and adjusted for liver disease history and smoking history.

^§^FDR-corrected *p*-values.

### Haplotype analysis

A reconstructed LD plot of the *NR1I3* gene, which has been generated utilizing the Haploview 4.2 software was shown in Supplementary Figure S1, and the results indicate that SNPs rs2502805 and rs10157822 were in linkage. According to the frequency distribution of haplotypes, the haplotypes with frequencies that were too low were excluded, and multivariate conditional logistic regression analysis was conducted on the haplotypes with frequencies greater than 2%. There was no significant difference in the haplotype distribution between the case and control groups (*p* > 0.05) (Table 3).

**TABLE 3:** Haplotype frequencies of *NR1I3* gene in two groups and the risks of AT-DILI.

Haplotype	AT-DILI cases (%)	Controls (%)	OR (95%CI)^a^	*p*-value
rs2502805-rs10157822				
C-C	61.4	57.9	1.000	
T-T	32.5	36.5	0.663–1.041	0.112
C-T	6.1	5.6	0.194–2.272	0.907

*NR1I3*, nuclear receptor subfamily 1 group I member 3; AT-DILI, antituberculosis drug-induced liver Injury; OR, odds ratio; 95% CI, 95% confidence interval.

^a^ Conditional logistic regression analysis and adjusted for liver disease history and smoking history.

### Subgroup analysis

The associations between SNPs and AT-DILI by sex are shown in Table 4. Utilizing multivariate conditional logistic regression analysis, we found that female patients carrying the T alleles of rs11584174 and rs2502805 in the *NR1I3* gene exhibited an elevated risk of AT-DILI (dominant model, adjusted OR = 1.386, 95% CI: 1.028–1.870, *p* = 0.032; dominant model, adjusted OR = 1.431, 95% CI: 1.058–1.935, *p* = 0.020, respectively). In addition, female patients harboring the TT allele of rs2502805 in the *NR1I3* gene displayed a heightened risk of AT-DILI (additive model, adjusted OR = 1.409, 95% CI: 1.024–1.940, *p* = 0.035). Marginally significant differences were also found in male patients carrying the GT/TT alleles of rs56163822 in the *NR1H4* gene (dominant model, adjusted OR = 1.196, 95% CI: 0.999–1.433, *p* = 0.051). Additionally, male patients carrying the T allele of rs11584174 in the NR1I3 gene displayed a decreased risk of AT-DILI (dominant model, adjusted OR = 0.793, 95% CI: 0.663–0.949, *p* = 0.011). However, after FDR correction, the difference was insignificant (*p* > 0.05).

**TABLE 4:** Subgroup analysis of sex with different genetic models and the risks of AT-DILI.

Gene	SNPs	Models	Male (N = 820)			Female (N = 300)		
			*OR* (95%CI)^a^	*p*-value	*p*-value^b^	*OR* (95%CI) ^a^	*p*-value	*p*-value^b^
*NR1I3*	rs10157822(T>C)							
	TT	Dom	0.788(0.493–1.258)	0.318		0.718(0.293–1.764)	0.470	
	TC	Rec	0.940(0.648–1.363)	0.744		0.627(0.350–1.123)	0.117	
	CC	Add	0.958(0.804–1.141)	0.630		1.181(0.873–1.598)	0.280	
	rs11584174(C>T)							
	CC	Dom	0.793(0.663–0.949)	0.011	0.066	1.386(1.028–1.870)	0.032	0.192
	CT	Rec	0.905(0.675–1.213)	0.504		1.329(0.697–2.534)	0.387	
	TT	Add	0.816(0.681–0.976)	0.026	0.156	1.320(0.967–1.801)	0.080	
	rs2307424(G>A)							
	GG	Dom	0.930(0.770–1.123)	0.450		1.065(0.771–1.472)	0.702	
	GA	Rec	1.028(0.837–1.262)	0.793		0.899(0.668–1.211)	0.483	
	AA	Add	0.922(0.776–1.095)	0.355		1.160(0.867–1.552)	0.319	
	rs2502805(C>T)							
	CC	Dom	1.119(0.939–1.334)	0.209		1.431(1.058–1.935)	0.020	0.120
	CT	Rec	0.994(0.782–1.265)	0.964		1.126(0.677–1.874)	0.648	
	TT	Add	1.121(0.940–1.337)	0.204		1.409(1.024–1.940)	0.035	0.210
*NR1H4*	rs56163822(G>T)							
	GG	Dom	1.196(0.999–1.433)	0.051		1.007(0.757–1.339)	0.964	
	GT	Rec	1.116(0.887–1.405)	0.348		0.860(0.581–1.273)	0.451	
	TT	Add	1.149(0.937–1.410)	0.181		1.105(0.807–1.514)	0.533	

*NR1I3*, nuclear receptor subfamily 1 group I member 3; *NR1H4*, nuclear receptor subfamily 1 group H member 4; AT-DILI, antituberculosis drug-induced liver Injury; SNPs, single nucleotide polymorphisms; OR, odds ratio; 95% CI, 95% confidence interval; Dom, dominant model; Rec, recessive model; Add, additive model.

a Conditional logistic regression analysis and adjusted for liver disease history and smoking history.

b FDR-corrected *p*-values.

### Comparisons of liver function indicators among patients with different genotypes

The correlations between liver function indicators of participants and different genotypes are shown in Table 5, Supplementary Table S3. The peak levels of ALT, AST and TBil in all patients with the *NR1H4*-rs56163822-GG genotype were notably greater than those in patients with the TT genotype (*p* = 0.011, *p* = 0.021, and *p* = 0.009, respectively). Additionally, this analysis revealed a noteworthy significant difference in the peak value of AST between the GG and GT genotype groups (34.0 U/L vs. 31.0 U/L, *p* = 0.007). Further subgroup analysis of rs56163822 in *NR1H4* also revealed significant differences in the peak values of ALT and AST among male patients (*p* = 0.002 and *p* = 0.019, respectively).

**TABLE 5:** Distribution of serum liver function in the peak value among different genotypes.

SNPs	Serum liver function^a^	Genotype				*p*-value^*#*^		
		AA	Aa	Aa	*p*-value^*^	*AA* vs. *Aa*	AA vs. aa	*Aa* vs. *aa*
rs11584174(C>T)	ALT	26.0(17.0–44.5)	27.0(16.2–70.6)	26.5(17.7–40.5)	0.598			
	AST	32.0(24.1–51.0)	33.0(24.0–60.8)	31.0(24.0–48.3)	0.595			
	TBil	14.4(10.7–19.6)	15.3(11.2–21.0)	16.0(11.9–21.4)	0.057			
	DBil	5.1(3.6–7.2)	5.2(3.5–7.4)	5.1(3.4–7.5)	0.815			
	ALP	96.9(79.3–121.0)	94.5(76.0–121.0)	87.5(74.0–115.5)	0.119			
rs2502805(C>T)	ALT	28.0(17.0–49.1)	25.0(16.5–45.3)	26.0(15.5–46.0)	0.282			
	AST	32.0(24.0–58.0)	32.0(25.0–51.5)	32.0(23.5–60.5)	0.899			
	TBil	14.9(10.8–20.6)	14.7(11.1–20.1)	15.4(11.4–21.4)	0.692			
	DBil	5.0(3.5–7.3)	5.1(3.7–7.2)	5.4(3.7–7.6)	0.596			
	ALP	96.0(78.0–124.0)	95.6(77.0–119.0)	94.0(78.0–116.0)	0.205			
rs56163822(G>T)	ALT	27.1(18.0–82.3)	26.0(16.0–40.0)	23.0(15.0–42.9)	0.020	0.058	0.011	0.439
	AST	34.0(25.0–65.0)	31.0(23.0–43.5)	30.6(23.6–50.2)	0.008	0.007	0.021	0.993
	TBil	15.5(11.2–21.0)	15.0(10.8–20.7)	13.7(10.8–18.5)	0.029	0.192	0.009	0.200
	DBil	5.1(3.5–7.3)	5.0(3.5–7.2)	5.3(3.9–7.7)	0.474			
	ALP	93.0(77.0–122.0)	96.0(81.0–122.0)	96.8(79.0–117.4)	0.617			

SNPs, single nucleotide polymorphisms; ALT, alanine transaminase; AST, aspartate transaminase; TBil, total bilirubin; DBil, direct bilirubin; ALP, alkaline phosphatase; AA, homozygous genotype of the wild type; Aa, heterozygous genotype; aa, homozygous genotype of the mutant type.

^a^ Values are presented as median (interquartile range).

*Differences in peak levels of each liver function index between different genotypes (Kruskal–Wallis test).

^#^Differences in peak levels of each liver function index between the two genotypes (Wilcoxon rank sum test).

### Online eQTL analysis

From the GTEx portal, further online eQTL analysis of NR1H4 (SNP rs56163822) and NR1I3 (SNP rs10157822, rs11584174, rs2307424 and rs2502805) genes was conducted with the gene expression in 208 liver samples, and the result revealed that significant differences in the expression levels of the three genotypes of NR1H4 rs56163822 in liver samples (*p* = 0.022), whereas no significant differences were observed in the expression levels of the three genotypes of NR1I3 in liver samples (*p* > 0.05) in Supplementary Figure S2.

## Discussion

This research indicated that genetic variants in NR1H4 might affect the susceptibility to AT-DILI, which further corroborates the hypothesis that perturbation of BA homeostasis causes liver injury.

The *NR1I3* gene, situated at the 1q23.3 locus on the human chromosome, comprises nine exons and encodes a member of the nuclear receptor superfamily and regulates ectobiotic and endobiotic metabolism. Distinct from many nuclear receptors, this transcriptional regulator remains constitutively active in the absence of a ligand, yet its activity is modulated by both agonists and inverse agonists. Upon ligand binding, this protein undergoes translocation to the nucleus to activate or repress the transcription of target genes. The ligands encompass bilirubin, various foreign compounds, steroid hormones, and prescribed medications like RIF and efavirenz (Petros et al., 2022). CAR and Th17 cells may be potential targets for novel therapeutic strategies for xenobiotic-induced liver inflammation (Shi et al., 2022). The indirect activation of the CAR/PXR, FXR, and Nrf2 pathways provides important molecular mechanisms in PFOA-induced human hepatotoxicity (Murase et al., 2023). Furthermore, the PXR and CAR pathways were activated in the PPARα^−/−^ mice, which may explain the lipid accumulation and hepatomegaly observed in the livers of mice exposed to PFDMO2HpA/PFDMO2OA (Ren et al., 2024).

Studies have illuminated the involvement of rs2307424 in the metabolism of efavirenz (Wyen et al., 2011). A meta-analysis revealed that the TT genotype of rs2307424 is associated with a lower concentration of efavirenz compared to the CC genotype. The TT genotype may exhibit linkage disequilibrium, which can lead to increased CAR and CYP2B6 activities. It increases the efficiency of metabolic enzymes and transporters, thereby reducing efavirenz concentrations (Ayuso et al., 2019), and rs2307424 is also associated with increased virological response (de Almeida Velozo et al., 2022). The homozygous rs10157822 variant genotype is correlated with a decreased risk of hyperbilirubinemia among female neonates (OR = 0.44, 95% CI: 0.20–0.95, *p* = 0.034) (Cheung et al., 2015), which may be due to acceleration of bilirubin transport by the homozygous variant. However, one case-control study failed to detect any association between six SNPs of CAR and risk of AT-DILI (Wang et al., 2019). Rs2502805 and rs11584174 are transcription factor-binding sites, and the Regulome DB score of rs11584174 is less than 1. However, no notable linkage was detected between rs11584174 and AT-DILI susceptibility, which may be due to differences in the distribution of gene frequencies in the population. The distribution of rs11584174 in the control population of this study was not consistent with the HWE, and there is no study on these two SNPs and related diseases, which can be further explored in different populations and larger sample sizes.

The *NR1H4* gene, which is situated on human chromosome 12q23.1 and comprises 17 exons, encodes FXR, a member of the nuclear receptor superfamily and a ligand-induced transcription factor. As a receptor for BAs, FXR binds to DNA and modulates the expression of genes that are integral to the synthesis and transportation of BAs (Yang et al., 2007; Attinkara et al., 2012; Kim et al., 2017). The suppression of the BA receptor FXR by pathogenic influences disrupts the downstream expression of BA synthesis and transport proteins, leading to abnormal functioning (He et al., 2024). In turn, this ultimately leads to elevated BA levels within the liver and intestine. Certain BA are toxic and induce inflammation at specific concentrations; whereas others can induce oxidative damage by interacting with cell membranes and fostering the production of reactive oxygen species (He et al., 2024). FXR reportedly participates in liver regeneration, which is mediated by hepatocyte proliferation, by regulating cell cycle progression, and *NR1H4* gene expression is significantly upregulated in severe liver injury (Cai et al., 2021). FXR also functions as a proviral host factor for hepatitis B virus (Mouzannar et al., 2019) and hinders liver fibrosis (Wang et al., 2018). Studies have shown that FXR agonists have antifibrotic and portal pressure-lowering effects in animal models; therefore, genotypes that lead to the suppression of FXR (such as rs56163822) expression elevate the risk of hepatic decompensation and death; however, no significant correlation was observed between the rs56163822 genetic polymorphism and the development of advanced chronic liver disease (Semmler et al., 2019). A study has shown that the rs56163822 GT genotype enhances the likelihood of developing spontaneous bacterial peritonitis among cirrhotic patients exhibiting ascites (Lutz et al., 2014).

This is the first study to explore the potential linkage between *NR1H4* and AT-DILI. This study revealed significant differences in the peak values of the serum liver function indicators ALT, AST and TBil among all patients with the GG and GT/TT genotypes of rs56163822 (27.1 U/L vs. 26.0 U/L vs. 23.0 U/L, *p* = 0.020; 34.0 U/L vs. 31.0 U/L vs. 30.6 U/L, *p* = 0.008; 15.5 μmol/L vs. 15.0 μmol/L vs. 13.7 μmol/L, *p* = 0.029, respectively). The present study also revealed significant differences in the peak values of ALT and AST among male patients with the three genotypes of rs56163822 (29.0 U/L vs. 26.9 U/L vs. 22.6 U/L, *p* = 0.002; 34.0 U/L vs. 32.0 U/L vs. 30.5 U/L, *p* = 0.019, respectively). Previous studies have revealed that median plasma BA concentrations are greater in men than in women (Frommherz et al., 2016; Xiang et al., 2012). The present results suggested that male patients carrying the *NR1H4*-rs56163822-GG may be more prone to liver injury, possibly because the G allele can affect the function of *NR1H4*, resulting in the disruption of BA homeostasis and increasing the risk of liver injury. Furthermore, the eQTL online database showed that the gene expression of SNP rs56163822-GG genotype was significantly higher than that of other genotypes (*p* = 0.022), further confirming the research findings at the mRNA level. If due to certain specific conditions or reasons of the patient, the NR1H4 gene site can be tested to determine whether there is a genetic variation. If the test finds that the patient carries this variation, the doctor can advise the patient to use other anti-tuberculosis drugs instead of those that may cause liver damage, so as to avoid the increased risk of liver injury when the patient uses certain drugs. However, a larger cohort study is needed to verify this conclusion.

The pivotal strength of this research is that it was a 1:4 individual-matched case‒control design with a considerable sample size in a Chinese population. This approach, coupled with comprehensive factor matching and multivariate logistic regression, effectively mitigates the influence of confounding variables potentially associated with AT-DILI. The substantial sample size further enhances statistical power and lays a solid foundation for in-depth subgroup analysis. Additionally, each AT-DILI patient strictly underwent a stringent causality assessment, minimizing the risk of misclassification bias. However, there are several limitations that ought to be acknowledged. Primarily, the rs2307424, rs11584174 and rs56163822 SNPs did not adhere to the HWE, which may be attributed to the nature of the study’s participant pool, where all controls were TB patients. Secondly, our participants were recruited from outpatient departments of four hospitals designated in Jiangsu Province, China, potentially limiting the generalizability of our findings to the entire Chinese population. Furthermore, we did not collect data on patients with previous hepatitis C infection or HIV infection. While the prevalence of these infections is relatively low in China, they are well-documented risk factors for hepatotoxicity.

## Conclusion

The present study is the first to undertake the initial evaluation of the potential correlations between genetic variations in *NR1I3* and *NRIH4* and the risk of AT-DILI among Chinese population. Our 1:4 case‒control design, with individual matching, suggests that the SNP rs56163822 in the *NR1H4* gene could be implicated in the vulnerability to AT-DILI in Chinese patients receiving anti-TB therapy. However, to substantiate these initial observations, additional research encompassing larger and more diverse populations is imperative.

## Data Availability

The raw data supporting the conclusions of this article will be made available by the authors, without undue reservation.
