# Supplementary material for: Roles of NR1I3 and NR1H4 polymorphisms in the susceptibility to antituberculosis drug-induced liver injury in China: a case‒control study
Source: Front Genet. 2024 Oct 23;15:1428319. doi: 10.3389/fgene.2024.1428319 (PMC11541836; doi:10.3389/fgene.2024.1428319)
Supplement: Supplementary file 1 [file Table1.docx]

Table S1 STROBE Statement—Checklist of items that should be included in reports of **case-control studies**.

|  | **Item No** | **Recommendation** | **Page** |
| --- | --- | --- | --- |
| **Title and abstract** | 1 | (*a*) Indicate the study’s design with a commonly used term in the title or the abstract | 1-2 |
|  |  | (*b*) Provide in the abstract an informative and balanced summary of what was done and what was found |  |
| **Introduction** | | |  |
| Background/rationale | 2 | Explain the scientific background and rationale for the investigation being reported | 3-4 |
| Objectives | 3 | State specific objectives, including any prespecified hypotheses | 4 |
| **Methods** | | |  |
| Study design | 4 | Present key elements of study design early in the paper | 4 |
| Setting | 5 | Describe the setting, locations, and relevant dates, including periods of recruitment, exposure, follow-up, and data collection | 4 |
| Participants | 6 | (*a*) Give the eligibility criteria, and the sources and methods of case ascertainment and control selection. Give the rationale for the choice of cases and controls | 4-5 |
|  |  | (*b*) For matched studies, give matching criteria and the number of controls per case |  |
| Variables | 7 | Clearly define all outcomes, exposures, predictors, potential confounders, and effect modifiers. Give diagnostic criteria, if applicable | 5 |
| Data sources/ measurement | 8* | For each variable of interest, give sources of data and details of methods of assessment (measurement). Describe comparability of assessment methods if there is more than one group | 5 |
| Bias | 9 | Describe any efforts to address potential sources of bias | 6 |
| Study size | 10 | Explain how the study size was arrived at | 4 |
| Quantitative variables | 11 | Explain how quantitative variables were handled in the analyses. If applicable, describe which groupings were chosen and why | 6 |
| Statistical methods | 12 | (*a*) Describe all statistical methods, including those used to control for confounding | 6 |
|  |  | (*b*) Describe any methods used to examine subgroups and interactions |  |
|  |  | (*c*) Explain how missing data were addressed |  |
|  |  | (*d*) If applicable, explain how matching of cases and controls was addressed |  |
|  |  | (*e*) Describe any sensitivity analyses |  |
| **Results** | | |  |
| Participants | 13* | (a) Report numbers of individuals at each stage of study—eg numbers potentially eligible, examined for eligibility, confirmed eligible, included in the study, completing follow-up, and analysed | 6-7 |
|  |  | (b) Give reasons for non-participation at each stage |  |
|  |  | (c) Consider use of a flow diagram |  |
| Descriptive data | 14* | (a) Give characteristics of study participants (eg demographic, clinical, social) and information on exposures and potential confounders | 6-7 |
|  |  | (b) Indicate number of participants with missing data for each variable of interest |  |
| Outcome data | 15* | Report numbers in each exposure category, or summary measures of exposure | 6-7 |
| Main results | 16 | (*a*) Give unadjusted estimates and, if applicable, confounder-adjusted estimates and their precision (eg, 95% confidence interval). Make clear which confounders were adjusted for and why they were included | 6-7 |
|  |  | (*b*) Report category boundaries when continuous variables were categorized |  |
|  |  | (*c*) If relevant, consider translating estimates of relative risk into absolute risk for a meaningful time period |  |
| Other analyses | 17 | Report other analyses done—eg analyses of subgroups and interactions, and sensitivity analyses | 7 |
| **Discussion** | | |  |
| Key results | 18 | Summarise key results with reference to study objectives | 8 |
| Limitations | 19 | Discuss limitations of the study, taking into account sources of potential bias or imprecision. Discuss both direction and magnitude of any potential bias | 9 |
| Interpretation | 20 | Give a cautious overall interpretation of results considering objectives, limitations, multiplicity of analyses, results from similar studies, and other relevant evidence | 8-9 |
| Generalisability | 21 | Discuss the generalisability (external validity) of the study results | 9 |
| **Other information** | | |  |
| Funding | 22 | Give the source of funding and the role of the funders for the present study and, if applicable, for the original study on which the present article is based | 10 |

*Give information separately for cases and controls.

Table S2 Information of five SNPs in NR1I3 and NR1H4 genes.

| Genes | SNPs | Position | MAF (%)^a^ | MAF (%)^b^ | Location^c^ | Alleles | SNPinfo^d^ | | | RegulomeDB^e^ | HWE  *p*-value^f^ |
| --- | --- | --- | --- | --- | --- | --- | --- | --- | --- | --- | --- |
|  |  |  |  |  |  |  | TFBS | Splicing | miRNA |  |  |
| NR1I3 | rs2307424 | 1:161232815 | 47.6 | 48.1 | synonymous variant | G>A | -- | Y | -- | 4 | **0.015** |
|  | rs2502805 | 1:161240051 | 31.1 | 36.4 | 2kb upstream variant | C>T | Y | -- | -- | 5 | 0.463 |
|  | rs10157822 | 1:161242119 | 36.9 | 40.9 | regulatory region variant | T>C | Y | -- | -- | 4 | 0.970 |
|  | rs11584174 | 1:161242663 | 19.9 | 25.9 | regulatory region variant | C>T | Y | -- | -- | 1f | **<0.001** |
| NR1H4 | rs56163822 | 12:100493323 | 12.6 | 34.3 | non-coding transcript variant | G>T | -- | Y | -- | 7 | **<0.001** |

NR1I3, nuclear receptor subfamily 1 group I member 3; NR1H4, nuclear receptor subfamily 1 group H member 4; SNPs, single nucleotide polymorphisms; MAF, minor allele frequency; UTR, untranslated regions; HWE, Hardy-Weinberg; TFBS, transcription factor binding sites; miRNA, micro ribonucleic acid.

^a^ MAF for Han Chinese in Beijing in the Hapmap database;

^b^ MAF for cases and controls;

^c^ SNP position in NCBI dbSNP (http://www.ncbi.nlm.nih.gov/projects/SNP);

^d^ https://snpinfo.niehs.nih.gov/;

^e^ http://www.regulomedb.org/;

^f^ HWE *p*-value in the controls.


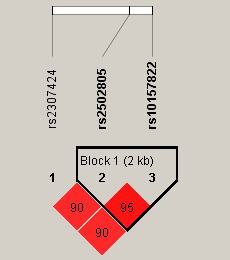


Figure S1 Linkage disequilibrium (LD) region composed of three SNPs in NR1I3 gene

This LD plot was generated with Haploview4.2 software. Each box shows D’ value correlation coefficient r^2^ values between the two SNPs, use white to red said correlation size, the larger the r^2^ the deeper the red color, the stronger the correlation.

TableS3 Distribution of serum liver function among different genotypes of SNP rs56163822 in NR1H4 gene under different sex.

| Serum liver  function | Genotypes | male | |  | female | |
| --- | --- | --- | --- | --- | --- | --- |
|  |  | Median (IQR) | *p*-value^*^ |  | Median (IQR) | *p*-value^*^ |
| ALT | GG | 29.0(19.7-91.0) | **0.002** |  | 23.6(15.0-39.3) | 0.885 |
|  | GT | 26.9(17.0-41.0) |  |  | 25.0(15.0-37.0) |  |
|  | TT | 22.6(15.0-40.8) |  |  | 24.0(16.0-48.0) |  |
| AST | GG | 34.0(25.1-64.0) | **0.019** |  | 32.0(24.0-72.8) | 0.248 |
|  | GT | 32.0(24.0-45.0) |  |  | 30.0(22.0-41.0) |  |
|  | TT | 30.5(23.9-48.8) |  |  | 31.0(23.0-53.0) |  |
| TBil | GG | 16.0(11.7-21.1) | 0.130 |  | 14.4(10.0-19.4) | 0.137 |
|  | GT | 15.5(11.2-21.0) |  |  | 13.9(9.8-19.6) |  |
|  | TT | 14.3(11.1-19.5) |  |  | 12.9(9.3-17.4) |  |
| DBil | GG | 5.2(3.7-7.4) | 0.530 |  | 4.7(3.4-7.1) | 0.629 |
|  | GT | 5.2(3.6-7.5) |  |  | 4.3(3.1-6.6) |  |
|  | TT | 5.5(4.0-7.9) |  |  | 4.8(3.2-6.3) |  |
| ALP | GG | 95.0(77.6-124.0) | 0.400 |  | 88.0(71.0-117.0) | 0.982 |
|  | GT | 99.1(82.0-124.8) |  |  | 89.0(76.0-106.0) |  |
|  | TT | 98.0(81.9-115.9) |  |  | 84.5(68.0-128.0) |  |

SNPs, single nucleotide polymorphisms; ALT, alanine transaminase; AST, aspartate transaminase; TBIL, total bilirubin; DBIL, direct bilirubin; ALP, alkaline phosphatase.

^*^ Differences in peak levels of each liver function index between different genotypes (Kruskal-Wallis test).


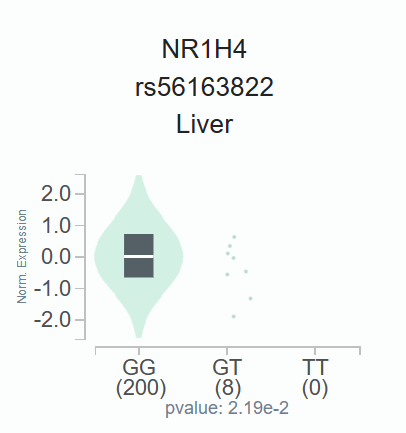

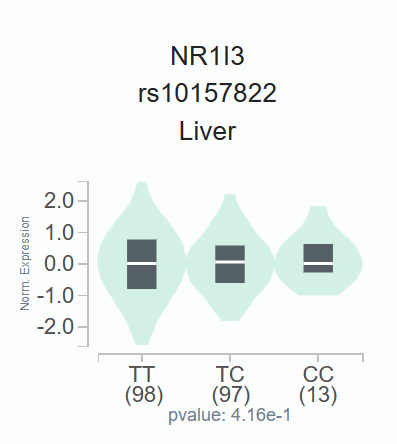

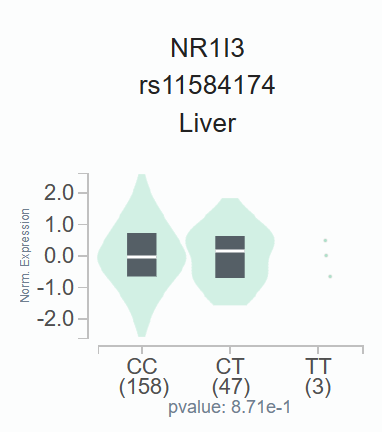

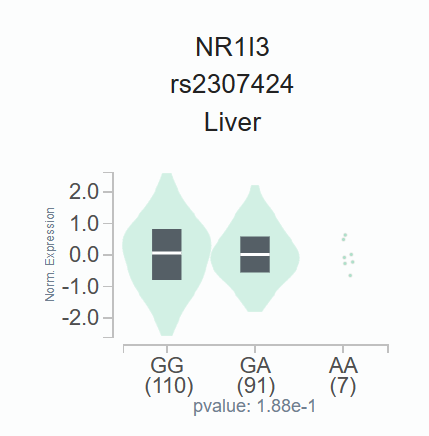

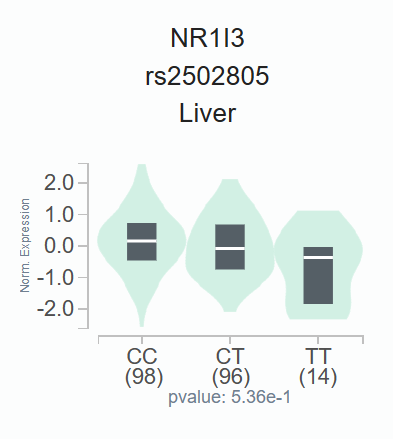


Figure S2. The Expression Quantitative Trait Loci (eQTL) violin plot of NR1H4 and NR1I3 gene expression in 208 liver samples. According to the eQTL violin plots, the vertical axis is the level of gene expression and horizontal axis is three different genotypes. The result revealed that significant differences in the expression levels of the three genotypes of NR1H4 rs56163822 in liver samples (p=0.022), whereas no significant differences were observed in the expression levels of the three genotypes of NR1I3 in liver samples (p>0.05). NR1H4: nuclear receptor subfamily 1 group H member 4; NR1I3: nuclear receptor subfamily 1 group I member 3; SNP: single nucleotide polymorphism.
